# The Study of HEMs Based on the Mechanically Activated Intermetallic Al_12_Mg_17_ Powder

**DOI:** 10.3390/molecules25163561

**Published:** 2020-08-05

**Authors:** Sergei Sokolov, Alexander Vorozhtsov, Vladimir Arkhipov, Ilya Zhukov

**Affiliations:** Laboratory of Metallurgy Nanotechnologies, National Research Tomsk State University, Lenin Avenue, 36, 634050 Tomsk, Russia; abv1953@mail.ru (A.V.); leva@niipmm.tsu.ru (V.A.); gofra930@gmail.com (I.Z.)

**Keywords:** alloys, mechanical activation, propellants, aluminum, magnesium, magnalium, metals combustion, high-energy materials, ignition, reactive materials

## Abstract

In this work, Al–Mg intermetallic powders were characterized and obtained by melting, casting into a steel chill and subsequent mechanical activation in a planetary mill. The method for producing Al_12_Mg_17_ intermetallic powder is presented. The dispersity, morphology, chemical composition, and phase composition of the obtained powder materials were investigated. Certain thermodynamic properties of high-energy materials containing the Al-Mg powder after mechanical activation of various durations were investigated. The addition of the Al-Mg powders to the high-energy composition (synthetic rubber SKDM-80 + ammonium perchlorate AP + boron B) can significantly increase the burning rate by approximately 47% and the combustion heat by approximately 23% compared with the high-energy compositions without the Al-Mg powder. The addition of the Al_12_Mg_17_ powder obtained after 6 h of mechanical activation provides an increase in the burning rate by 8% (2.5 ± 0.1 mm/s for the mechanically activated Al_12_Mg_17_ powder and 2.3 ± 0.1 mm/s for the commercially available powder) and an increase in the combustion heat by 3% (7.4 ± 0.2 MJ/kg for the mechanically activated Al-Mg powder and 7.1 ± 0.2 MJ/kg for the commercially available powder). The possibility of using the Al-Mg intermetallic powders as the main component of pyrotechnic and special compositions is shown.

## 1. Introduction

Improving the energy characteristics of high-energy materials (HEMs), improving traditional components, and searching for new components is a relevant objective for many fields of science and technology. High-energy metal powders began to be intensively studied as fuels for burning systems when Russian scientists Kondratyuk and Tsander suggested using high-energy metals (Al, Be, B, Mg, Zr, Ti, etc.) as energy additives for propellants [1]. Metal powders with a high enthalpy of combustion are of great interest as reactive materials in the compositions of propellants, explosives, pyrotechnics, etc [2]. Fine metal powders that are part of high-energy systems are mainly used as components to control the burning rate and reduce ignition delays [3,4,5].

Aluminum powder is the most affordable, widely studied, and effective additive in various high-energy compositions [4,6,7]. Despite the extensive use of aluminum as a metal fuel, there are certain disadvantages. The major disadvantage is the formation of an oxide film on the surface of aluminum particles, which prevents the rapid flow of chemical reactions and forms agglomerates before ignition [8]. To reduce agglomeration, aluminum particles must be optimized for rapid ignition. There are various methods to solve this problem: the use of nanosized aluminum powder [9]; the use of metallic and polymer coatings [10,11]; the use of other additives [12,13]; and the replacement of pure aluminum powder with powders of intermetallic alloys [14,15,16,17]. Despite the attractive properties of nanosized aluminum powder, it can reduce the specific impulses of propellants, lead to poor rheology and loss of quality during aging [18], since it can contain 10–25 wt % of aluminum oxide [7]. Metallic and polymer coatings result in decreased agglomeration sizes in comparison to pure Al, but they are still about five times larger than the initial particle size. Additives (such as nickel) require large amounts to reduce agglomeration, but higher molecular weight products can significantly reduce performance. Al-based alloys allow a more efficient use of the base material and expand its scope. Various metals are used to change characteristics. The addition of alloying materials (Mg, Zr, Ti, etc.) in aluminum leads to an increase in the combustion heat and burning rate and a reduction in the ignition delay compared with pure aluminum powder. Al-based intermetallic alloys (Al-Mg, Al–Zr, Al–Ti, etc.) are of great interest as metal fuel in various energy compositions [2,3,4,5,6,7,8,9,10,11,12,13,14,15,16,17,18,19,20]. In particular, Al-Mg alloys have long been studied as reactive materials and as replacements for pure metal powders [14,15,16,17,21,22]. The use of Mg powders in compositions of high-energy materials has several advantages. Compositions with magnesium powder ignite at a lower temperature than with aluminum powder and can be used to ignite compositions with a high ignition temperature [23].

One of the promising, simple, and inexpensive methods of obtaining metal powders with increased reactivity is mechanical activation in a planetary mill. The first studies in this field began in the 19th century [24]. Using the mechanical method, there is the milling of the material in a solid or liquid state without changing the chemical composition. During the milling, powders are affected by the high energies, multiple impacts, abrasion, crushing, and shearing actions of milling bodies on the walls of the ball mill container and the material. The high strain rate related to these collisions results in a combination of fracture, ductility, atomic mixing, and a partial reaction between the components of the material [25]. Using this method, it is possible to obtain mechanically activated powders with improved physics and chemical properties, which are very difficult or impossible to obtain by other methods [26,27]. There are studies reporting the effect of mechanical activation on the thermal-physical properties of metal powders [27,28].

Despite the considerable interest in investigating the effect of the mechanical activation of eutectic alloys on the combustion parameters of high-energy materials, quantitative data on their combustion temperature, burning rate, oxidation degree, and other important properties remain limited. In particular, there are no studies on the effect of the duration and mode of mechanical activation on the combustion parameters of the Al-Mg system as a part of high-energy additives.

Thus, the purpose of this work is to experimentally study the structure, dispersity, oxidation degree, burning rate, and combustion heat of the Al-Mg powder materials in the high-energy compositions, depending on the duration of mechanical activation in a planetary mill.

## 2. Materials and Methods

### 2.1. Preparation of Al-Mg Material

First, 99% pure Al-Mg alloy was obtained from commercial-purity aluminum ingot (99 wt % Al, 0.9 wt % Si, 0.04 wt % Mg and other impurities) and commercial-purity magnesium ingot (99.95 wt % Mg, 0.01 wt % Al, 0.01 wt % Mn, and other impurities). The alloy was fabricated by casting in a clay-graphite crucible. The fusion of aluminum and magnesium was carried out in argon. Aluminum ingot was loaded into the crucible and placed in a furnace. When the temperature reached 730 °C, Al melt was removed from the furnace, and magnesium ingot was added into the aluminum melt in small parts in a mass ratio of Al:Mg—1:1. The magnesium ingots were shaped cubes and parallelepipeds, and the approximate mass of individual fragments was 100–150 g. For homogeneous distribution of the components in the melt, mixing was carried out using the device with a rotation speed of 1500 rpm [29]. Mechanical mixing was carried out until the magnesium ingot was completely dissolved in the aluminum melt. The resulting alloy was poured into a steel chill at a temperature of 670 °C.

### 2.2. Mechanical Activation

**Figure 1:**
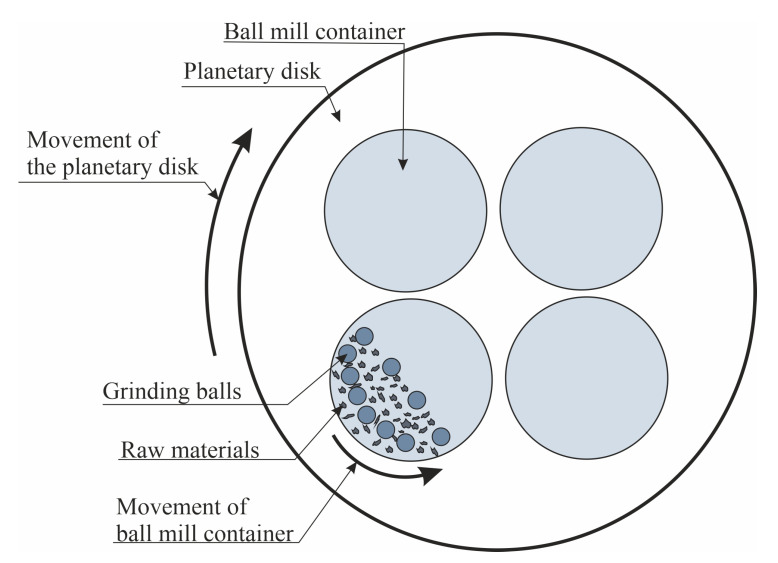
Schematic diagram of the planetary mill.

The obtain alloy was crushed using a jaw crusher. The obtain Al-Mg flakes were mechanically milled in a planetary mill (Figure 1), the main element of which is the ball mill container. The lid of the container has two taps. One of them is used to evacuate the working space, and the second is used to pump inert gas (argon). Steel balls with a diameter of 8.7 mm were used as grinding bodies. The mass ratio of grinding bodies to powder mixture was 2:1. The mechanical activation was conducted for 6 h in an argon atmosphere with a rotation frequency of 12 rpm.

### 2.3. Particle Size, Chemical Analysis, Morphology, and Phase Composition

The average particle size of the mechanically activated powder mixtures was measured using an ANALYSETTE 22 MicroTec plus apparatus (Fritsch GmbH, Dresden, Germany) based on the laser diffraction method in the range 0.08–2000 µm and the Fraunhofer theory. The dispersion was carried out in a liquid medium (distilled water). The chemical analysis of the intermetallic Al-Mg powders was performed using an XRF-1800 wavelength-dispersive spectrometer. When calibrating the XRF-1800, a curve was constructed showing the relationship between the element content in the standard sample and its measured intensity (calibration curve); then, the element content in the studied sample was found. The microstructure of the powders was determined using a Vega3 Tescan (TESCAN ORSAY HOLDING, Brno, Czech Republic). To determine the amount of oxygen in the powder, a LECO ONH (LECO Corporation, St. Joseph, MI, USA) analyzer was used. X-ray diffraction analysis of the powders was performed using a Shimadzu XRD 6000 diffractometer with CuKα radiation. The phase composition was determined using the POWDER CELL 2.4 program and PDF 4+ (Powder Diffraction File) database.

### 2.4. Calorimetric Studies

Calorimetric studies of the mechanically activated powder mixtures were performed using thermogravimetric analysis (TGA) and differential scanning calorimetry (DSC) on a Mettler Toledo thermal analyzer with a TGA/SDTA 851 module (Mettler-Toledo, LLC, Columbus, OH, USA) in Al_2_O_3_ crucible in the temperature range of 25–1200 °C (heat rate 10 K/min) in an O_2_/Ar atmosphere.

### 2.5. Process to Obtain Samples of HEMs

The basic composition of the samples contained the following: bidispersed ammonium perchlorate (AP) with a dispersion of 160–315 µm and less than 50 µm in a ratio of 60/40 as an oxidizing agent; divinyl rubber based on butadiene rubber, which was plasticized with transformer oil in a ratio of 20/80 (SKDM-80) as a combustible binder; and amorphous black boron powder (B-99) as an energy additive. The amorphous boron was selected due to its high combustion heat (Q_boron_ = 58 KJ/g). In traditional high-energy materials, aluminum powder is used as an energy additive with a lower combustion heat (Q_Al_ = 31 KJ/g). The Al_12_Mg_17_ powder additive was selected to increase the combustion completeness of boron. Aluminum powder has a high combustion temperature, and magnesium is a highly flammable component. The content of components in the studied samples (Table 1) was selected from the condition of ensuring the same value of the excess coefficient of the oxidizer (α = 0.5), where BC—Basic composition; BC(0)—Basic composition with the addition of Al-Mg without mechanical activation (t = 0); BC(2)—Basic composition with the addition of Al-Mg with mechanical activation (t = 2 h); BC(6)—Basic composition with the addition of Al-Mg with mechanical activation (t = 6 h).

From the obtained mixtures, the samples with a diameter of 10 mm and a height of 30–40 mm were pressed. The obtaining of the studied samples consisted of sequential mixing of the components (ammonium perchlorate, Al-Mg powders, and processing aids) with a polymer combustible binder. To obtain high-quality samples, the oxidizer charge was divided into several parts. For homogeneous distribution of the components, each charge was mixed for 10–15 min.

In this work, the Al-Mg powder, because of its high exothermicity, was used as a catalytic additive that is capable of increasing the energy parameters of boron in the high-energy compositions and also significantly increasing their burning rate. Experimental studies to determine the combustion heat and burning rate, as well as the calculation of the combustion heat, were carried out according to the approved methods presented in [30,31].

### 2.6. Study of the Combustion Heat and Burning Rate of HEMs

The combustion heat was measured using a calorimetric apparatus, the main part of which is a bomb calorimeter. To obtain results with high accuracy, important requirements are imposed on the bomb calorimeter described in [32]. Critical requirements are that it is gastight and has mechanical strength under high pressure. The container of the calorimetric apparatus was filled with 3 L of distilled water, and a bomb calorimeter was immersed in it. For a uniform and quick distribution of water temperature throughout the calorimetric apparatus, a propeller was placed next to the bomb calorimeter. The propeller provided rotation at a speed of 400–500 rpm. The water temperature was measured using a Beckman metastatic thermometer, which allows high-precision measurements. After performing all the above steps, the samples of HEMs were ignited, and the temperature of water in the container of the calorimetric apparatus was measured. Three experiments were performed for each composition.

The burning rate of the studied samples was measured in the air atmosphere under a pressure of 0.1 MPa. The initial temperature of the samples is the ambient temperature (22 °C). The samples with a diameter of 10 mm and a height of 30 mm were armored with a linoleum solution in acetone. The samples were ignited by the local heating of the upper surface with a molybdenum spiral. Burning time was fixed by a stopwatch. The process was observed visually and also with a NIKON D600 camera and a Yukon thermal imager, UX 70P (Yukon Advanced Optics Worldwide, UAB, Lithuania).

## 3. Results and Discussion

### 3.1. Particle Size, Chemical Analysis, Morphology, and Phase Composition

**Figure 2:**
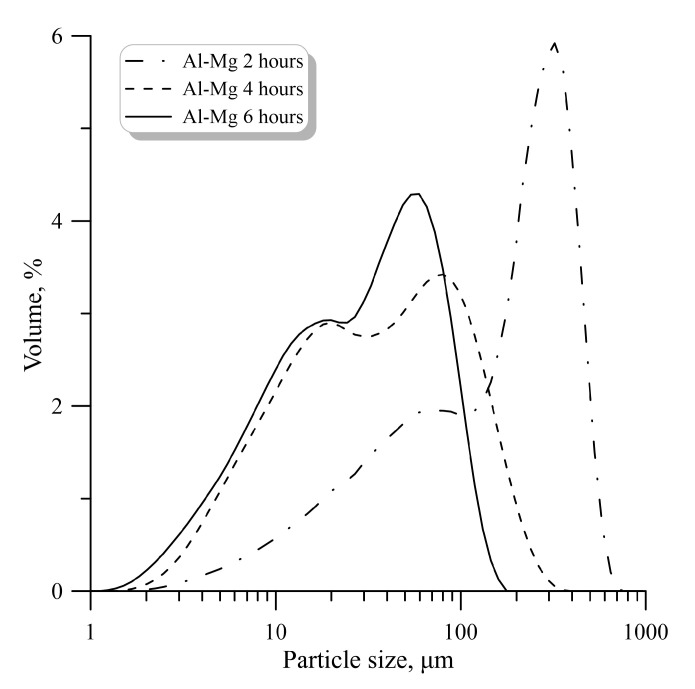
Particle size distribution for the Al-Mg powder.

**Figure 3:**
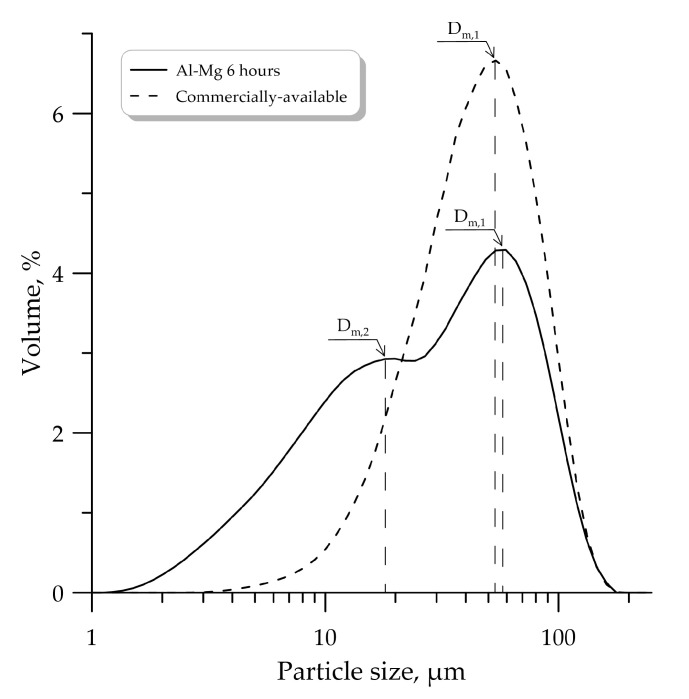
Particle size distribution for the Al-Mg powder and the commercially available Al-Mg powder.

The particle size distribution for the Al-Mg powder after mechanical activation in the planetary mill is shown in Figure 2. The particle size distribution substantially depends on the duration of mechanical activation. With an increase in the duration of mechanical activation, the particle size of the Al-Mg powder decreases. The obtained Al-Mg powder (after 6 h of mechanical activation) was compared with commercially available Al-Mg powder. It was found that the average particle size is 49 μm for the obtained Al-Mg powder and 50 μm for the commercially available Al-Mg powder, respectively (Figure 3), where D_m,1_ and D_m,2_—modal particle diameter.

**Figure 4:**
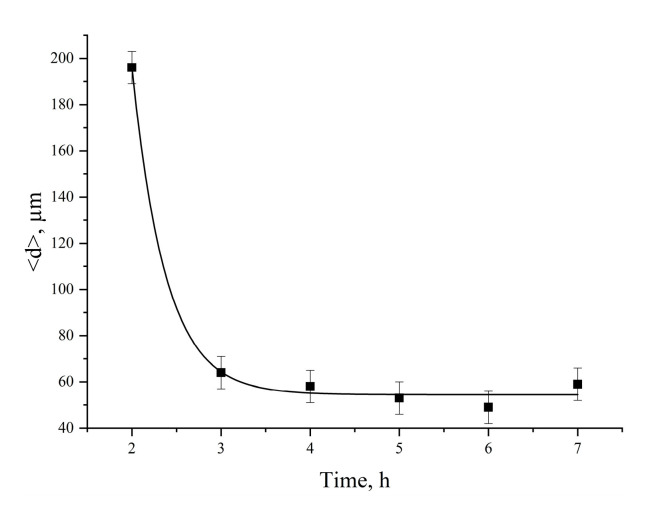
Dependence of the average particle size of the Al-Mg powder on the duration of mechanical activation.

The dependence of the average particle size <d> of the Al-Mg powder on the duration of mechanical activation in the planetary mill is shown in Figure 4. After 2 h of mechanical activation, the average particle size of the Al-Mg powder decreases significantly from 196 μm (2 h) to 64 μm (3 h).

Al-Mg powders (after mechanical activation) have a bimodal particle size distribution (2 maxima D_m,1_ and D_m,2_ are observed in Figure 2; Figure 3). The mass median diameter (d_43_) for the unimodal distribution function of the commercially available powder and the bimodal distribution function of the mechanically activated powder are approximately the same (the difference is approximately 1 μm). However, the bimodal distribution function (the presence of two fractions) can significantly affect the characteristics of the studied processes.

X-ray fluorescence analysis of the obtained Al-Mg alloy is shown in Table 2. The Al-Mg alloy obtained by casting has an almost equilibrium content of the main components with minimum oxygen content. Quantitative analysis of the oxygen content in the Al-Mg alloy and the mechanically activated Al-Mg powder was performed. It was found that the oxygen content in the Al-Mg alloy before mechanical activation is approximately 0.003 wt %, while the oxygen content in the Al-Mg powder after mechanical activation does not exceed 0.1 wt %.

Aluminum powder is known to have a dense oxide film on the surface. The oxide film of the intermetallic Al_12_Mg_17_ powder is not as dense as that of aluminum powder. This is due to the protective properties of oxide films. The possibility of the formation of such a film is determined by the condition of continuity formulated by Pilling and Bedworth (Pilling-Bedworth coefficient (PBC)) [33].

(1) $$PBC_{metal}=\frac{Oxide\;volume}{Metal\;volume}$$

According to the condition of continuity, the molecular volume of the oxide formed from the metal and oxygen should be greater than the molecular volume of the metal used to form the oxide molecule. Otherwise, the oxide film is not enough to cover the entire metal with a continuous layer. As a result, the oxide film is loose and porous. If PBC > 1, the film forms under compression conditions; therefore, the film is continuous and may have protective properties. If PBC < 1, the film in the process of its growth sustains tension, which contributes to its destruction and cracking. In this case, the film is not continuous (e.g., the oxide film of magnesium). The effect of magnesium on the oxide film is enhanced, since the magnesium content in the film is always many times higher than the magnesium content in the particle [34]. If the magnesium content is greater than 1% in the alloy, the oxide film consists entirely of magnesium oxide MgO [34]. The PBC coefficient for some metal oxides is shown in Table 3.

**Figure 5:**
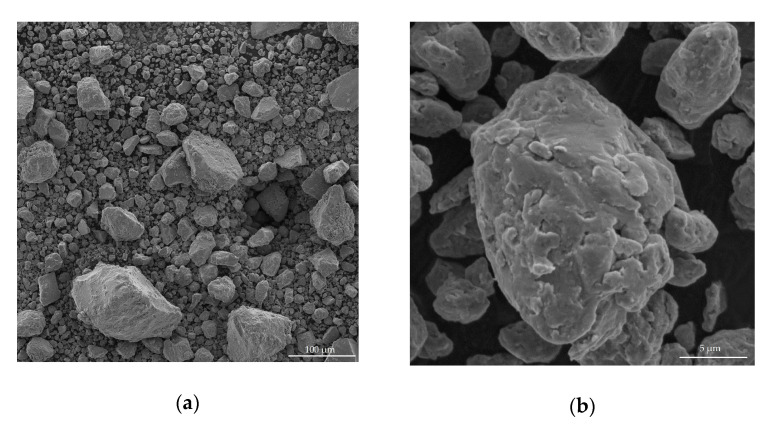
SEM-images (Det. SE+BSE) of the AlMg powder after 6 h of mechanical activation at magnifications × 509 (**a**) and × 10200 (**b**)**.**

The SEM images of the Al-Mg powder after 6 h of mechanical activation are shown in Figure 5. Figure 5a,b are identical, but they have different magnifications (Figure 5a magnification is equal × 509, and Figure 5b magnification is equal × 10200). In particular, Figure 5a shows a wide range of powder particle sizes, and Figure 5b shows in more detail the complex shape and structure of the particles. The powder has separate particles of irregular shape, which is typical for materials after mechanical activation.

**Figure 6:**
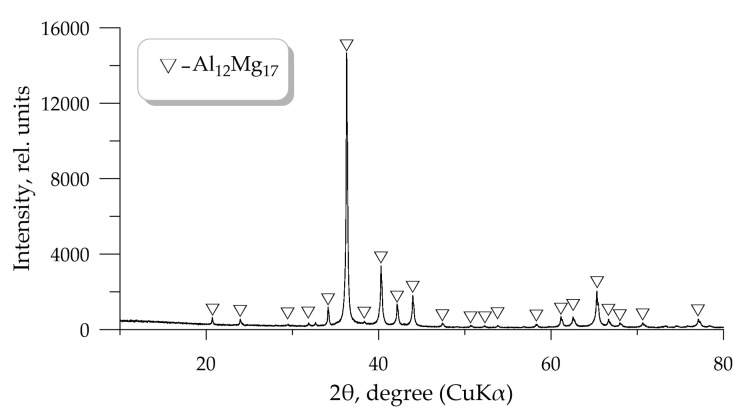
XRD pattern of the Al-Mg powder after 6 h of mechanical activation.

The XRD pattern of the mechanically activated Al-Mg powder is shown in Figure 6. It was found that peaks in the XRD pattern corresponds to the Al_12_Mg_17_ phase. According to the Al-Mg state diagram, the Al_12_Mg_17_ phase is formed at the same percentage (Al 50%/Mg 50%) of each component in the alloy, which is confirmed by the results of chemical analysis (Table 2). It should also be noted that the XRD patterns of the mechanically activated Al-Mg powder and commercially available Al-Mg powder are completely identical.

### 3.2. Calorimetric Studies

**Figure 7:**
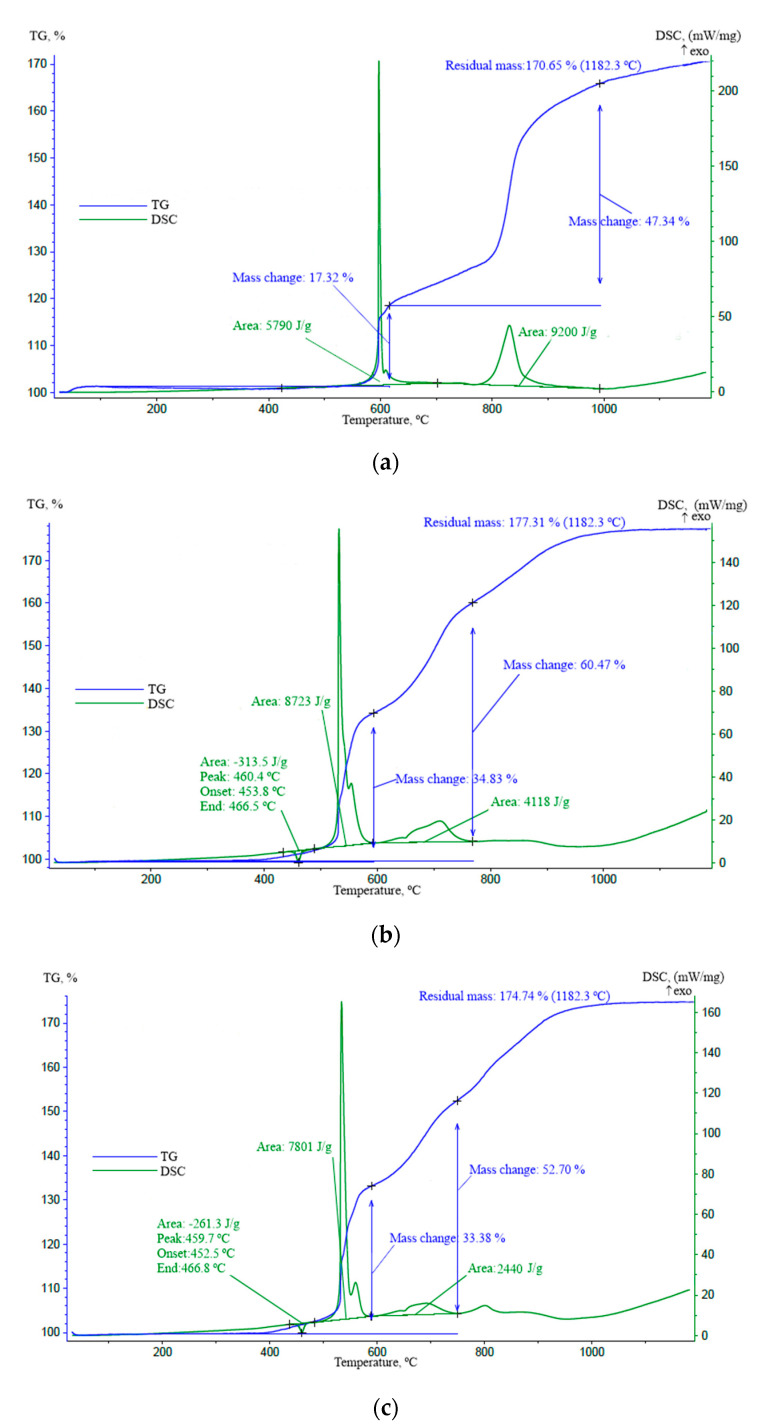
TG/DSC curves of the Al-Mg powder obtained after 2 h (**a**), 4 h (**b**) and 6 h (**c**) of mechanical activation.

The results of thermogravimetric analysis (TGA) and differential scanning calorimetry (DSC) of the Al-Mg powder after 2, 4, and 6 h of mechanical activation are shown in Figure 7. As can be seen from TG/DSC curves, the Al-Mg powder after 2 h of mechanical activation does not show endothermic melting. The Al-Mg powders after 4 h and 6 h of mechanical activation show endothermic melting, which corresponds to the melting point of 450 °C and is consistent with the Al-Mg state diagram [35].

The oxidation degree of the powder obtained after 2 h of mechanical activation is 70.65% at two exothermic peaks at temperatures around 600 °C and 820 °C. The oxidation degree of the powder obtained after 4 h of mechanical activation is 77.31% at four exothermic peaks at temperatures around 520 °C and 710 °C. The oxidation degree of the powder obtained after 6 h of mechanical activation is 74.4% at exothermic peaks at temperatures around 520 °C and 690 °C. The oxidation degree of the powders obtained after 4 and 6 h of mechanical activation is close to the theoretical value for the case of complete oxidation (77.4%), which indicates the formation of Al_2_O_3_ and MgO [16]. According to the results of thermal analysis, with an increase in the time of mechanical activation, the exothermic peak shifts to the zone of lower temperatures, and mechanical activation for more than 4 h does not significantly affect the position of peaks on the TG/DSC curves of the studied materials.

**Figure 8:**
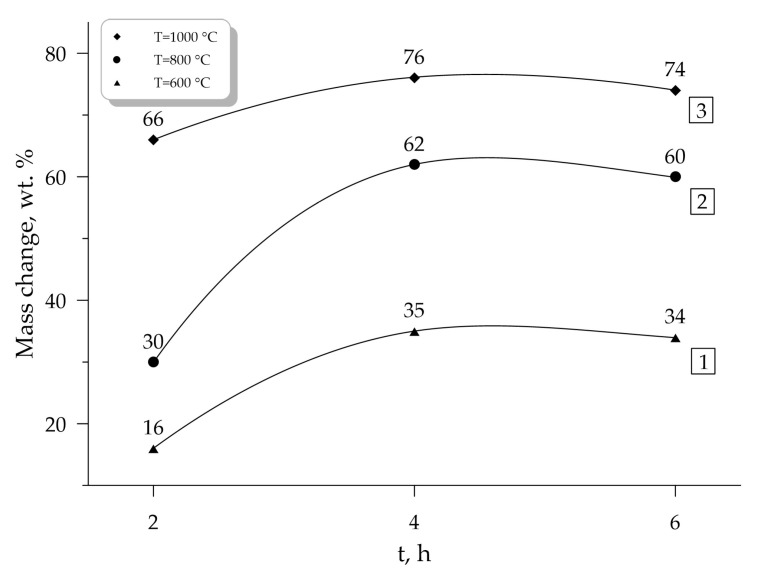
Dependence of mass change on the mechanical activation duration at different temperatures (1—T = 600 °C, 2—T = 800 °C, 3—T = 1000 °C).

The summarized results of the obtained experimental data of thermogravimetric analysis (TGA) and differential scanning calorimetry (DSC) are presented in Table 4, as well as the results of mass changes depending on the mechanical activation duration are shown in Figure 8.

### 3.3. Combustion Heat and Burning Rate

**Figure 9:**
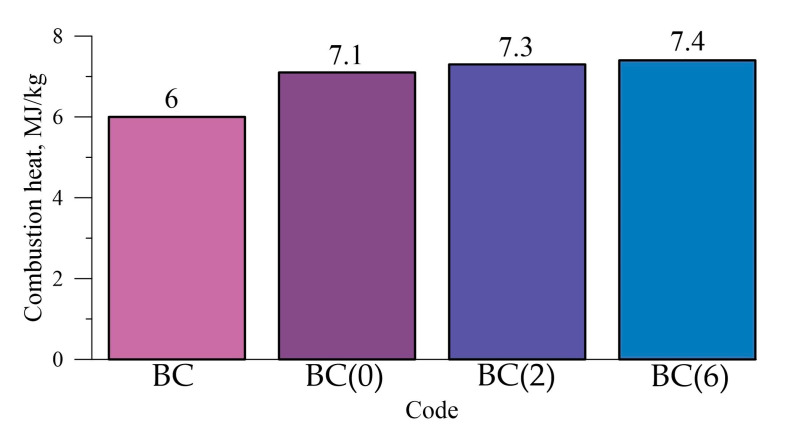
Combustion heat of the HEM compositions.

The combustion heat was determined for the samples whose compositions are shown in Table 1. As can be seen from Figure 9, the addition of the mechanically activated Al-Mg powder to the composition of HEMs leads to an increase in the combustion heat by approximately 23% compared with the composition without the mechanically activated Al-Mg powder. It was found that the addition of the mechanically activated Al-Mg powder to the composition BC (basic composition), regardless of the duration of mechanical activation and dispersion of the powder, does not affect the combustion heat. Comparison of the studied Al-Mg powder after 6 h of mechanical activation and commercially available Al-Mg powder showed that the difference in the combustion heat is 3% (7.4 ± 0.2 MJ/kg for the mechanically activated Al-Mg powder and 7.1 ± 0.2 MJ/kg for the commercially available powder). From experimental data, it was found that the combustion heat of the compositions of HEMs does not depend on the method of obtaining powders and is the same for all compositions containing the Al-Mg powders. As can be seen from Table 2 and Figure 9, during mechanical activation, there are no changes in the chemical composition of the material, which would contribute to an increase in the combustion heat.

**Figure 10:**
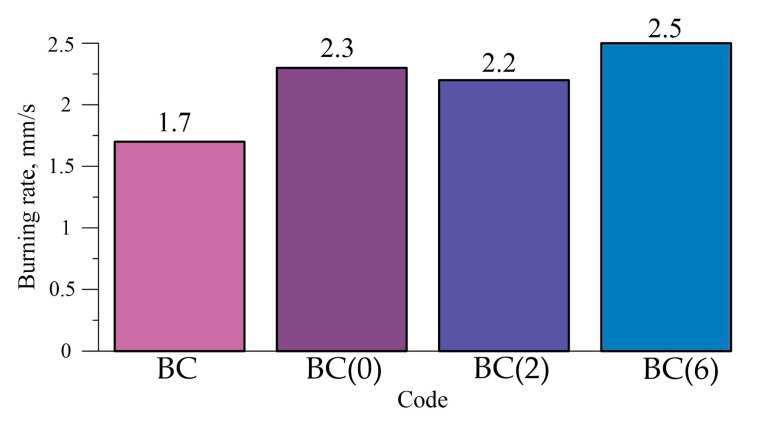
Burning rate of the HEM compositions.

The results of the burning rate of the prepared compositions of HEMs (Table 1) in the air at normal pressure (Figure 10) were obtained. The sample containing only amorphous boron powder (composition BC) has the lowest burning rate, while when the Al-Mg powder was added to the composition, the burning rate of the composition increases by approximately 47%. At the same time, the Al-Mg powder obtained after 6 h of mechanical activation gives the largest increase in the burning rate. Comparison of the studied Al-Mg powder obtained after 6 h of mechanical activation and the commercially available Al-Mg powder showed that the difference in the burning rate is 8% (2.5 ± 0.1 mm/s for the Al-Mg powder obtained after 6 h of mechanical activation and 2.3 ± 0.1 mm/s for the commercially available powder).

The difference in the combustion modes is probably explained the difference in the structure of the Al-Mg powder particles and the formation of bimodal particle size distribution. The difference in the structure as well as formation of bimodal distribution are explained by the difference in the method for obtaining the Al-Mg powders. The commercially available Al-Mg powder was obtained by spraying inert gas in a sealed chamber. The studied Al-Mg powder was obtained by mechanical activation in the planetary mill, and the phase composition of the studied Al-Mg powder is dominated by the Al_12_Mg_17_ phase. The mechanically activated Al_12_Mg_17_ powder has increased reactivity and is easier to react due to structural yielding and the formation linear crystal defects: dislocations and point defects (ionic and atomic vacancies, interstitial ions) [36].

The summary results of the obtained experimental data are presented in Table 5, where Q—combustion heat; U—burning rate; ΔQ and ΔU—difference in the combustion heat and burning rate of the compositions with Al-Mg alloy from the base composition.

## 4. Conclusions

The Al-Mg powder, in view of its high exothermicity, was used as the catalytic additive that is capable of increasing the energy parameters of boron in the high-energy compositions and also significantly increasing their burning rate.

The addition of the mechanically activated Al_12_Mg_17_ powder to the composition (SKDM-80 + ammonium perchlorate + amorphous boron) leads to an increase in the burning rate and the combustion heat by approximately 47% and 24%, respectively. The greatest increase in the burning rate is achieved when using Al_12_Mg_17_ powder obtained after 6 h of mechanical activation in the planetary mill.

Comparison of the burning rate and the combustion heat of the Al-Mg powders obtained by various methods showed that the addition of the studied Al_12_Mg_17_ powder obtained after 6 h of mechanical activation provides an increase in the burning rate by 8% (2.5 ± 0.1 mm/s for the mechanically activated Al_12_Mg_17_ powder and 2.3 ± 0.1 mm/s for the commercially available powder) and the combustion heat by 3% (7.4 ± 0.2 MJ/kg for the mechanically activated Al_12_Mg_17_ powder and 7.1 ± 0.2 MJ/kg for the commercially available powder).

It was found that the addition of the Al-Mg powder in the compositions of HEMs, regardless of the method of obtaining and dispersion of the powders, does not affect a significant change in heat release.

The phase composition of the commercially available Al-Mg powder and intermetallic powder obtained by the alloying and subsequent mechanical activation in the planetary mill are represented by the fragile intermetallic Al_12_Mg_17_ phase, which according to the state diagram of the Al-Mg system corresponds to the eutectic region.

It was found that the increase in the duration of mechanical activation of the Al-Mg powder from 2 to 3 h leads to a significant decrease in particle size from 196 to 64 μm, and the morphology of the Al-Mg powder consists of individual particles of irregular shape, which is typical for powders after mechanical milling. It should be noted that Al-Mg powders (after mechanical activation) have a bimodal particle size distribution. The mass median diameter (d_43_) for the unimodal distribution function of the commercially available powder and the bimodal distribution function of the mechanically activated powder are approximately the same (the difference is 1 μm).

It was found that with an increase in the duration of mechanical activation in the planetary mill, the exothermic peak of the Al_12_Mg_17_ powder on the DSC curve shifts to the zone of lower temperatures, and mechanical activation for more than 4 h does not significantly affect the position of peaks on the TG/DSC curves of the studied materials.

## Figures and Tables

**Table 1:** The components of high-energy compositions.

Code	Components of HEMs, wt %					
	AP	Boron	SKDM-80	Commercially-Available Al-Mg	Al-Mg 2 h	Al-Mg 6 h
BC	74	15	11	-	-	-
BC(0)	70.5	14.3	10.5	4.7	-	-
BC(2)	70.5	14.3	10.5	-	4.7	-
BC(6)	70.5	14.3	10.5	-	-	4.7

**Table 2:** Results of X-ray fluorescence analysis of the Al-Mg alloy.

Element	Result, at. %	
	Al-Mg Alloy	Commercially–Available Al-Mg Alloy
Al	50.4121	44.4907
Mg	48.6949	51.8649
Si	0.5359	0.0473
Fe	0.2229	0.0275
Cr	0.0595	0.0284
Ti	0.0250	-
C	0.0249	-
Zn	0.0094	0.0052
Ni	0.0076	0.0111
Ga	0.0076	0.0075
O	0.0001	3.5126
Cu	-	0.0040
Mo	-	0.0007
Nb	-	0.0001

**Table 3:** Pilling-Bedworth coefficient (PBC) for some metals.

Metal	Si	Ti	Zr	Al	Mg	Ca	Li
PBC	2.04	1.73	1.45	1.45	0.81	0.64	0.58

**Table 4:** Summary of the results of TG/DSC analysis.

	2 h	4 h	6 h
Endothermic peak, °C	-	460.4	459.7
Endothermic peak area, J/g	-	−313.5	−261.3
Exothermic peak 1, °C	600	520	520
Exothermic peak 2, °C	820	710	690
Exothermic peak area 1, J/g	5790	8723	7801
Exothermic peak area 2, J/g	9200	4118	2440
Mass change (600 °C), wt %	16	35	34
Mass change (800 °C), wt %	30	62	60
Mass change (1000 °C), wt %	66	76	74
Residual mass (1182.3 °C), wt %	70.65	77.31	74.74

**Table 5:** Summary of the results of combustion heat and burning rate. BC: Basic composition; BC(0): Basic composition with the addition of Al-Mg without mechanical activation (t = 0); BC(2): Basic composition with the addition of Al-Mg with mechanical activation (t = 2 h); BC(6): Basic composition with the addition of Al-Mg with mechanical activation (t = 6 h).

Code	d_43_, μm	D_m,1_, μm	D_m,2_, μm	Q, MJ/kg	ΔQ, %	U, mm/s	ΔU, %
BC	-	-	-	6.0 ± 0.2	-	1.7 ± 0.08	-
BC(0)	50	54	-	7.1 ± 0.2	19	2.3 ± 0.08	36
BC(2)	196	322	80	7.3 ± 0.2	22	2.2 ± 0.08	27
BC(6)	49	58	18	7.4 ± 0.2	23	2.5 ± 0.08	47
